# Perceptions, Barriers, and Facilitators of Provider-Initiated and Voluntary HIV Testing and Counseling Among Health Care Workers: Protocol for a Multicenter Cross-Sectional Study

**DOI:** 10.2196/69832

**Published:** 2025-12-22

**Authors:** Bingyi Wang, Leiwen Fu, Ke Liu, Cailing Ao, Simin He, Shilan Xie, Liwei Wan, Guoren Wang, Zhen Lu, Yong Lu, Fang Yang, Yan Li, Xiaobing Fu, Huihong Deng

**Affiliations:** 1 Institute of HIV/AIDS Control and Prevention, Guangdong Provincial Center for Disease Control and Prevention, Guangzhou, China; 2 Department of HIV/AIDS Control and Prevention, Guangdong Provincial Academy of Preventive Medicine, Guangzhou, China; 3 Luhe Center for Disease Control and Prevention, Shanwei, China; 4 Beijing Chest Hospital, Capital Medical University, Beijing, China; 5 Beijing Tuberculosis and Thoracic Tumour Research Institute, Beijing, China; 6 Department of Disease Surveillance, Guangzhou Baiyun District Center for Disease Control and Prevention, Guangzhou, China; 7 Institute of Noncommunicable Disease Control and Prevention, Guangdong Provincial Center for Disease Control and Prevention, Guangzhou, China; 8 School of Public Health (Shenzhen), Sun Yat-Sen University, Guangzhou, China; 9 School of Public Health, Guizhou Medical University, Guiyang, China; 10 General Office, Guangdong Provincial Center for Disease Control and Prevention, 160 Qunxian Road, Panyu District, Guangzhou, Guangdong, 511445, China

**Keywords:** HIV testing and counseling, HIV prevention strategy, health services accessibility, health care workers, China

## Abstract

**Background:**

HIV testing and counseling interventions have been pivotal in efforts to curb the HIV epidemic, with diverse delivery models implemented globally. However, existing studies primarily focus on individual perspectives, with limited attention given to the essential role of health care workers in the effective implementation of voluntary counseling and testing (VCT) and provider-initiated testing and counseling (PITC) services in China.

**Objective:**

This protocol describes the design of the Provider-initiated Views on PITC and VCT Study (PIVOT Study), which aims to assess health care workers’ perceptions, barriers, and facilitators related to the implementation of PITC and VCT in Guangdong Province, China.

**Methods:**

The PIVOT Study is a multicenter, cross-sectional observational study. Eligible participants are health care workers employed at various health care service institutions, including hospitals, VCT clinics, the Centers for Disease Control and Prevention, and community health centers. We will use a convenience sampling method. Data will be collected via a structured digital questionnaire covering 5 domains: sociodemographic information, general health status, psychosocial characteristics, knowledge related to PITC and VCT, and experiential insights regarding service provision. Descriptive statistics will be used to characterize variable distributions, and multivariable logistic regression models will assess associations between independent and outcome variables. Secondary analyses will explore subgroup differences based on age, years of experience, sex, institution type, and geographical location. A total of approximately 400 health care workers will be recruited.

**Results:**

The PIVOT Study proposal was submitted in December 2024 and received funding approval in May 2025, with official project initiation planned for July 2025. Study design and survey instrument revisions were completed between December 2024 and March 2025. A pilot survey was completed from April to May 2025, followed by questionnaire testing and refinement from June to August 2025. Formal data collection was conducted from September to November 2025, with data cleaning and preliminary analyses scheduled from December 2025 to January 2026. Final data analysis and manuscript preparation are planned from February to June 2026.

**Conclusions:**

The PIVOT Study will generate important insights into health care workers’ perspectives on PITC and VCT service delivery in China. The findings are expected to inform the development of targeted strategies to strengthen HIV testing efforts, particularly among underrepresented populations such as older adults. Study results will be disseminated through peer-reviewed journals and national and international conferences.

## Introduction

### Background

The Joint United Nations Programme on HIV/AIDS estimated that, as of 2023, approximately 39.9 million individuals worldwide were living with HIV [1]. In China, this number has risen from 0.65 million in 2005 to an estimated 1.1 million in 2021 [2,3]. The ongoing HIV/AIDS epidemic remains a pressing public health challenge, presenting significant obstacles for prevention and control efforts.

HIV testing and counseling interventions have been pivotal in efforts to curb the HIV epidemic, with diverse delivery models—such as voluntary counseling and testing (VCT) and provider-initiated testing and counseling (PITC)—targeted at reducing undiagnosed infections and addressing the needs of key populations affected by the epidemic [4-6]. VCT is conducted in clinical settings, with an emphasis on pre- and posttest counseling and upon obtaining informed consent from the individual [7]. In contrast, PITC, as a streamlined model, simplifies information gathering and counseling on high-risk behaviors, uses an opt-out system, and is integrated into routine health care services [8].

While some systematic evidence examines the effects of VCT and PITC on individuals’ behaviors, psychological well-being, and care, much of this research primarily focuses on individual outcomes [9-11]. Consequently, there is a notable lack of evidence regarding the essential role of health care workers in ensuring the effective implementation of VCT and PITC. Moreover, the successful implementation of HIV testing and counseling—an inherently complex process—depends on well-defined procedures, an in-depth understanding of protocols, and proficient communication skills among health care professionals [4]. However, how these factors influence the ability of health care workers to carry out VCT and PITC remains unanswered. If these factors are inadequately addressed, patients may experience decreased uptake of testing services, suboptimal counseling quality, increased psychological distress, and delayed linkage to appropriate care [12]. Previous studies have highlighted that failures in VCT and PITC delivery can reinforce HIV-related stigma and compromise health outcomes [13].

### Research Questions

To address these identified gaps, the Provider-initiated Views on PITC and VCT Study (PIVOT Study) was established to generate foundational evidence. This multicenter observational study aims to elucidate the current perceptions, barriers, and facilitators related to PITC and VCT among health care workers in China, along with their correlates. The primary and secondary research questions of the PIVOT Study are as follows.

- Primary research questions: What are the perceptions of PITC and VCT among health care workers? What are correlates of these perceptions? What barriers do health care workers encounter in the implementation of VCT and PITC?
- Secondary research questions: How can the effectiveness of PITC and VCT be enhanced from the perspective of health care workers? Based on health care workers’ perceptions and barriers, how can the effectiveness of PITC and VCT be enhanced? What specific support do health care workers need to improve the quality of PITC and VCT services?

This protocol outlines the study design, target populations, survey instruments, and procedural methodologies. It serves as a vital resource for researchers aiming to replicate the described cross-sectional approach and represents a valuable guide for those interested in facilitating a deeper understanding of the barriers and facilitators influencing PITC and VCT from the perspective of health care workers.

## Methods

### Ethical Considerations

During the planning phase, stakeholder meetings were convened, involving health care providers from various health care service institutions, social workers from the Guangdong Association of STD & AIDS Prevention and Control, and research team members to evaluate the feasibility of the PIVOT Study. All investigators and personnel received training in research ethics and the protection of human subjects (Multimedia Appendix 1). All procedures contributing to this work comply with the ethical standards of relevant national and institutional committees on human experimentation and with the Helsinki Declaration. The anonymity and confidentiality of the participants in this study will be ensured and maintained according to laws and regulations. Digital informed consent will be obtained from all participants before any procedure. The PIVOT Study has received preliminary ethics approval from the Human Research Ethics Committee of the Guangdong Provincial Center for Disease Control and Prevention (W96-027C-KS), which was granted to support the initial pilot study and funding application process. Following funding approval and confirmation that all data were fully anonymized and involved no individual-level identifiers in any datasets, transcripts, or publications, the study received a waiver of full ethical review, and the documentation of this waiver has been provided in Multimedia Appendix 1.

### Dissemination

Findings from the PIVOT Study will be disseminated widely through peer-reviewed scientific journals and at national and international conferences.

### Study Design and Setting

The PIVOT Study is a multicenter, cross-sectional study focused on health care workers across various health care service institutions in Guangdong Province, including hospitals, VCT clinics, the Centers for Disease Control and Prevention, and community health centers. Using a convenience sampling method, the study aims to assess the current perceptions, barriers, and facilitators related to PITC and VCT from the perspective of these professionals. We did not predefine the number of participating institutions. Instead, we aimed to maximize diversity in health care service settings. The final number of participating institutions and geographical areas will be reported as part of the study results.

### Patient and Public Involvement

Patients and the public were not involved in the design, conduct, reporting, or dissemination plans of this research.

### Study Population

#### Eligibility Criteria

The eligibility criteria are presented in Textbox 1.

Textbox 1. **Eligibility criteria.**

**Inclusion criteria**

- Aged ≥18 years
- Worked in any health care service institution for at least 12 months
- Understands the survey instrument of the Provider-initiated Views on PITC and VCT Study (PIVOT Study)

**Exclusion criteria**

- Unable to provide informed consent
- Participated for less than 12 months in any health care institution
- Submitted incomplete or invalid survey responses

Sample Size

This study uses logistic regression, which informs the required sample size calculations. For logistic regression analysis, the final sample size is established as 10 to 15 times the number of covariates, guided by findings from previous research [14]. The study will assess 7 variables related to sociodemographic and general health conditions, 5 variables addressing psychosocial characteristics, 2 variables concerning perceptions of VCT or PITC implementation, and 3 variables focused on experiences with VCT or PITC implementation. This totals 17 variables, necessitating a minimum sample size of 255 participants, derived from the 15-fold multiplier. To enhance statistical power for subgroup analyses, we will recruit an additional 40% of participants. Consequently, the final sample size will be at least 360 participants. Accounting for potential missing data and ensuring validity, we will distribute a minimum of 400 questionnaires.

### Data Collection

Data collection will be conducted via a digital questionnaire encompassing 5 key components: sociodemographic information, general health status, psychosocial characteristics, knowledge related to the implementation of VCT or PITC, and experiential insights regarding these services. A convenience sampling approach will target health care workers within various health care institutions in Guangdong Province, spanning the period from September 2025 to November 2025. The notification package containing survey instructions and questionnaire QR codes will be coordinated by 2 trained research assistants in collaboration with provincial health authorities. These assistants will receive prior training covering ethical communication practices, technical procedures for QR code distribution, and standardized responses to participant inquiries.

Quality control measures will include real-time monitoring of submissions through the online dashboard with automated validation checks such as a minimum completion time threshold of 10 minutes, coupled with weekly progress updates to participating institutions. Before the commencement of the survey, the digital questionnaire will reiterate the study’s objectives. Participants will be adequately informed about the focus of the PIVOT Study and will electronically sign an informed consent form via the online platform. We will ensure the confidentiality of all participants’ private information and guarantee their right to withdraw from the study at any time.

### Survey Instrument

The design of the questionnaire and selection of variables were informed by key constructs from the theory of planned behavior (eg, attitudes, subjective norms, and perceived behavioral control) and the capability, opportunity, and motivation (COM-B) framework, both of which are commonly applied in implementation research. Multidimensional variables will be collected, encompassing sociodemographic factors (eg, age, sex, years of experience in health care, type of health care institution, and geographic location), health conditions (eg, general health and specific health issues), psychosocial characteristics (eg, depressive symptoms, feelings of loneliness, resilience, perceived stress, and job satisfaction), experiences with VCT or PITC implementation, perceptions regarding the implementation of VCT or PITC, and experiences with referral services. Given the ongoing paradigmatic evolution in the demographic landscape of individuals living with HIV in China—driven by increased accessibility to effective antiretroviral therapy [15,16], better management of coinfections and comorbidities [17], and a rising number of new infections among older adults [18,19]—the PIVOT Study also seeks to explore health care workers’ perspectives on this demographic, including their understanding of older adults’ sexual behaviors, their attitudes toward aging populations, and their views on sexual education targeted at older adults.

Given that older adults represent a population at potential risk for HIV infection, the PIVOT Study also tries to explore health care workers’ perspectives on this demographic, including their understanding of older adults’ sexual behaviors, attitudes toward older adults, and views on sexual education targeting older populations. Additionally, insights from the promotion of HIV testing will inform the implementation of hepatitis C virus (HCV) testing. Thus, we have also incorporated perceptions related to HCV testing. Specific variables within each domain are detailed in Table 1. Prior to the formal launch of the survey, the instrument was adjusted through a preliminary presurvey (pilot study). A preliminary pilot survey was conducted to assess face validity and content validity. The questionnaire was subsequently revised based on participant feedback. Formal evaluation of the instrument’s reliability and validity will be conducted upon completion of data collection.

**Table 1.** The survey instrument content of the Provider-initiated Views on PITC and VCT Study.

Domain	Variable
Sociodemographic	Not specifically listed
Health conditions	General health Specific health
Psychosocial characteristics	Depressive symptoms Loneliness symptoms Resilience Perceived stress Job satisfaction
Perceptions of VCT^b^ or PITC^c^ implementation	Channels of awareness for VCT Knowledge related to VCT implementation Number of VCT clinics known Channels of awareness for PITC Knowledge related to PITC implementation
Experience with VCT or PITC implementation	Experience with VCT implementation Reasons for failure of VCT implementation Experience with PITC implementation Approach to PITC implementation Reasons for failure of PITC implementation
Experience with referral services implementation for people living with HIV	Experience with referral service implementation for people living with HIV Number of referral services implementation for people living with HIV Willingness to implement referral services for people living with HIV
Perceptions of HCV^a^ testing implementation	Knowledge related to HCV Experience with HCV testing Approach to HCV testing implementation
Perceptions related to older adults	Attitudes toward older adults Sexual knowledge of older adults Attitudes toward sexual health education in older adults
Perceptions related to digital health in HIV/AIDS control and prevention	Attitudes toward digital health in HIV/AIDS control and prevention Perceived necessity of using digital health in HIV/AIDS control and prevention

a HCV: hepatitis C virus.

b VCT: voluntary counseling and testing.

c PITC: provider-initiated testing and counseling.

### Data Security and Data Management

#### Data Download

Data will be exported from the digital platform in CSV format upon the study’s closure and updated weekly to a secure institutional storage platform. According to the data security protocols, access to these data will be restricted to 3 administrators (BW, HD, and XF) from the PIVOT Study.

#### Data Security Protocols

The primary principle guiding data security is that all access to data is strictly on a need-to-know basis, a policy that permeates all aspects of data management throughout the study. All project-related data, including survey results and laboratory testing outcomes, will be stored on a secure file storage platform. To ensure the integrity of data handling, stringent protocols must be followed when accessing, storing, and downloading data under institutional guidelines. Given the sensitive nature of the data, particularly regarding work experiences with PITC and VCT, maintaining a high level of anonymity is paramount. The survey will remain anonymous.

### Statistical Analysis

Statistical analyses will be conducted using Python (version 3.8 with the following libraries: pandas [version 2.0.0], NumPy [version 1.24.2], and SciPy [version 1.10.1]; Python Software Foundation); SPSS (version 24.0; IBM Corp); and SAS (version 9.4; SAS Institute Inc). Descriptive statistics will characterize the distribution of variables, while multivariable logistic regression models will be used to assess the associations between independent variables and outcome variables. The assumptions for conducting logistic regressions will be checked and confirmed to be fulfilled, including multicollinearity, linearity, independence, and outliers [20]. Subgroup analyses will explore differences based on age, years of experience in health care, sex, type of health care institution (including hospitals, VCT clinics, Centers for Disease Control and Prevention, and community health centers), and geographic location (comparing the Pearl River Delta region to non-Delta areas). The statistical significance was set as *P*<.05.

## Results

**Figure 1.**
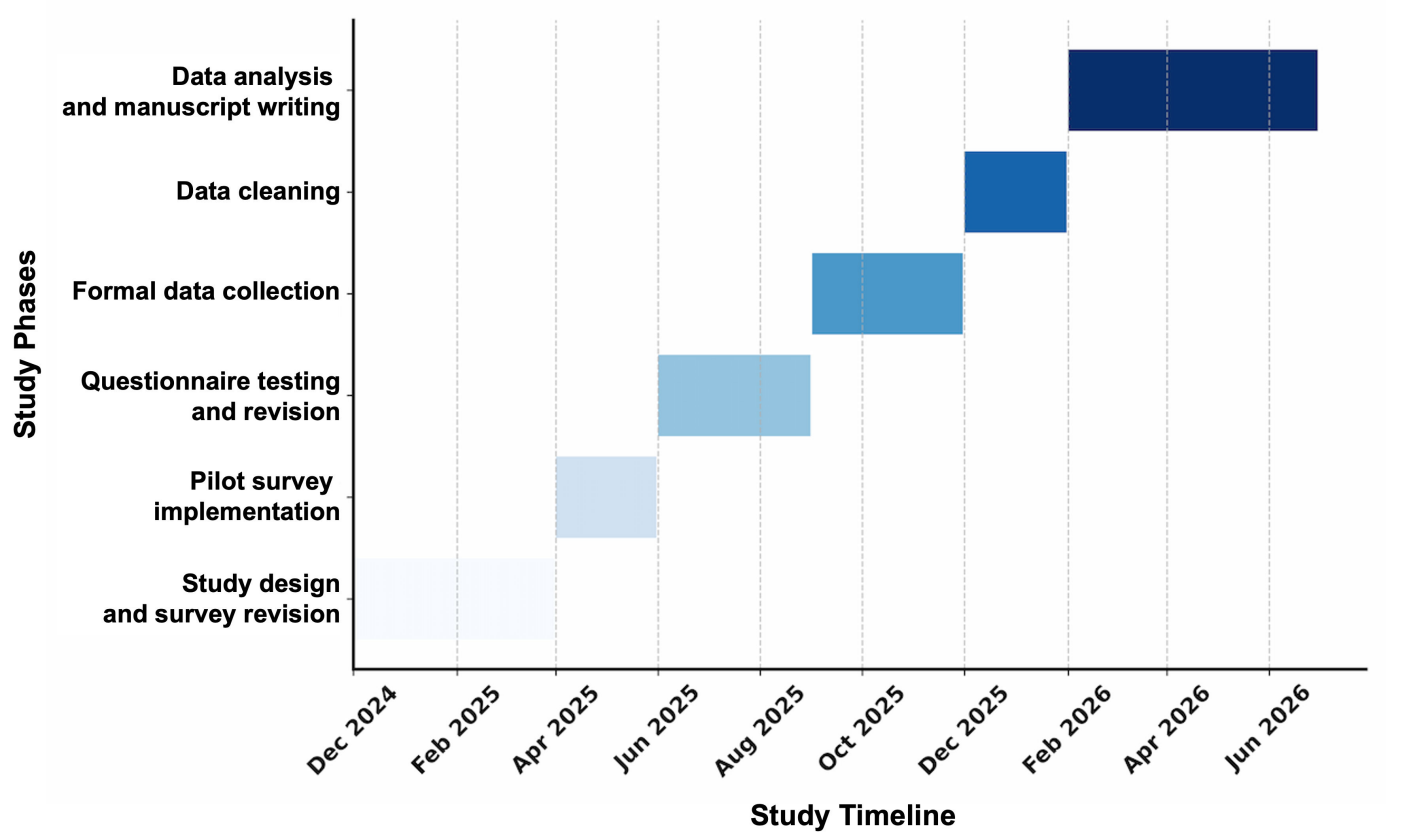
Anticipated study timeline for the Provider-initiated Views on PITC and VCT Study.

The PIVOT Study proposal was submitted in December 2024 and received funding approval in May 2025, with official project initiation planned for July 2025. Study design and survey instrument revision were conducted between December 2024 and March 2025. A pilot survey was conducted from April to May 2025, followed by questionnaire testing and refinement scheduled from June to August 2025. Formal data collection will be undertaken between September and November 2025, and data cleaning and preliminary analyses will be conducted from December 2025 to January 2026. Final data analysis and manuscript preparation are planned from February to June 2026. A visual representation of the anticipated study timeline is provided in Figure 1.

## Discussion

### Summary

Despite the essential role of health care workers in expanding HIV testing services, little is known about their decision-making processes and contextual barriers regarding PITC and VCT in China. The PIVOT Study addresses this gap by systematically comparing perceptions, implementation challenges, and contextual facilitators for PITC and VCT among frontline health care workers in diverse clinical settings. Findings from this study will inform context-specific strategies to improve provider engagement and optimize the integration of HIV testing approaches into routine care.

### Anticipated Findings

Recent epidemiological data in China have shown a notable increase in the incidence of HIV infections among older adults, highlighting an emerging public health challenge. However, existing prevention strategies have largely focused on younger populations, leaving a critical gap in addressing the unique needs of older individuals. By prioritizing older adults in the PIVOT Study, we aim to contribute to a more inclusive understanding of HIV prevention and emphasize the necessity of targeted interventions such as age-appropriate counseling, tailored risk communication, and community-based screening programs. The focus represents a timely response to shifting epidemiological trends and underscores the study’s relevance to contemporary public health needs.

To eliminate the HIV epidemic by 2030, the Joint United Nations Program on HIV/AIDS has set forth an ambitious global target known as the 95‐95‐95 strategy, aiming for 95% of the individuals living with HIV to be diagnosed, 95% of those diagnosed to receive sustained antiretroviral therapy, and 95% of those on therapy to achieve viral suppression by 2025 [21]. By the end of 2020, China reported progress rates of 79%, 93%, and 96% toward these targets, respectively [22]. Over the past 4 decades, China has achieved significant advancements in the prevention and control of HIV/AIDS, in line with the nation’s socioeconomic progress [3]. Nevertheless, there remains a notable gap in reaching the initial 95% target compared to other upper–middle-income countries [2]. Despite substantial annual investments in HIV/AIDS prevention and treatment, enhancing HIV testing efforts may require a focus on optimizing the allocation of these resources. Understanding health care workers’ perceived barriers and facilitators has been shown to be critical for improving the success of HIV testing initiatives and optimizing resource use [23,24].

Both VCT and PITC implementations are predominantly driven by individual willingness rather than a coordinated collective effort [7,10]. A lower willingness to engage with testing often translates to increased effort and cost in connecting high-risk groups with HIV testing and care services [25]. The lack of coordination, stemming from limited accessibility for high-risk populations, can result in disparities in HIV testing rates, ultimately impeding progress toward timely diagnosis and treatment [2]. Yet, much of the current research on PITC and VCT focuses solely on service recipients. In this context, the PIVOT Study could offer an essential perspective, aiming to assess health care workers’ perceptions of PITC and VCT and to identify barriers and facilitators impacting these initiatives from the viewpoint of these professionals. The findings of the PIVOT Study could provide a solid foundation for optimizing resource allocation in HIV testing and counseling interventions across various contexts. For instance, identifying barriers such as time limitations, stigma concerns, or lack of institutional support may guide the development of specific strategies, such as task-shifting models, stigma-reduction training, or decision-support tools embedded in routine clinical workflows.

Beyond its primary focus on HIV testing and counseling, the PIVOT Study also recognizes the broader implications for HCV elimination efforts. Given the high rates of HIV-HCV coinfection among key populations, strategies that enhance HIV testing uptake may simultaneously facilitate earlier HCV diagnosis and linkage to care. Strengthening PITC services could thus play a pivotal role in integrated infectious disease control programs, aligning with global health initiatives aimed at the elimination of viral hepatitis as a major public health threat.

This study offers several innovative contributions to the field. First, it highlights the critical importance of expanding HIV prevention efforts to older adults, a demographic increasingly affected by HIV but often underrepresented in public health interventions. Second, it underscores the potential for HIV testing strategies to contribute to broader infectious disease control initiatives, particularly in supporting HCV elimination efforts. Together, these aspects enhance the novelty, practical relevance, and public health significance of the PIVOT Study.

### Limitations

Our study also had some limitations. First, causality among the study variables cannot be concluded due to the cross-sectional design. Future longitudinal research is essential to elucidate the causal relationships among these variables. Second, the use of nonprobability sampling methods may limit the representativeness of our sample and introduce potential selection bias, as participants were not randomly selected. Third, we required participants to have at least 12 months of experience in HIV-related service provision to ensure that their perceptions and reported barriers were grounded in substantive professional practice. However, we acknowledge that this criterion may have excluded newer health care workers who could provide valuable insights from a fresh or evolving perspective. Fourth, the PIVOT Study was conducted in a province in southern China, where participation bias cannot be avoided, although multicenter studies have broadened representativeness. Guangdong Province, being economically developed and relatively successful in AIDS prevention and control, may not reflect the unique geographic and demographic challenges faced by other regions. Thus, findings may not be generalizable to provinces with different contexts. More experimental research in more provinces is needed to test our findings. Additionally, our sample size was estimated using a rule-of-thumb approach based on the number of predictors, without formal power or sensitivity analysis, due to the exploratory nature of the study and lack of prior pilot data. This may limit the statistical power, particularly for subgroup comparisons. Finally, the data from our study rely on self-reported information, which led to inevitable recall bias among participants.

## Supplementary material

10.2196/69832 Multimedia Appendix 1 Human Research Ethics Committee of Guangdong Center for Disease Control approval document.

## Abbreviations

COM-B: capability, opportunity, and motivation
HCV: hepatitis C virus
PITC: provider-initiated HIV testing and counseling
PIVOT: Provider-initiated Views on PITC and VCT Study
VCT: HIV voluntary counseling and testing

## References

[1] (2024). The urgency of now: AIDS at a crossroads. Joint United Nations Programme on HIV/AIDS (UNAIDS).

[2] He S, Dong W, Fairley CK (2024). Optimizing health resource allocation for improving timely HIV diagnosis in China. J Int AIDS Soc.

[3] Li H, Gao R, Zhang C (2024). Evolution of HIV/AIDS Prevention and Control Policies in China: a grounded theory approach. China CDC Wkly.

[4] Witzel TC, Lora W, Lees S, Desmond N (2017). Uptake contexts and perceived impacts of HIV testing and counselling among adults in East and Southern Africa: a meta-ethnographic review. PLoS ONE.

[5] Liu Y, Su R, Li D, Wang S, Han M (2024). Temporal and spatial trends in HIV positivity rate for VCT clinics - China, 2015-2022. China CDC Wkly.

[6] Hu Q, Xu J, Chu Z (2013). Barriers to acceptance of provider-initiated testing and counseling among men who have sex with men in Shenyang, China: a cross-sectional study. Biomed Res Int.

[7] Xu Z, Ma P, Chu M (2020). Understanding the role of voluntary counseling and testing (VCT) in HIV prevention in Nantong, China. Biomed Res Int.

[8] Wang QQ, Chen XS, Yin YP (2011). HIV/STD pattern and its associated risk factors among male STD clinic attendees in China: a foci for HIV intervention. BMC Public Health.

[9] Denison JA, O’Reilly KR, Schmid GP, Kennedy CE, Sweat MD (2008). HIV voluntary counseling and testing and behavioral risk reduction in developing countries: a meta-analysis, 1990-2005. AIDS Behav.

[10] Kennedy CE, Fonner VA, Sweat MD, Okero FA, Baggaley R, O’Reilly KR (2013). Provider-initiated HIV testing and counseling in low- and middle-income countries: a systematic review. AIDS Behav.

[11] Hensen B, Baggaley R, Wong VJ (2012). Universal voluntary HIV testing in antenatal care settings: a review of the contribution of provider‐initiated testing & counselling. Tropical Med Int Health.

[12] Inghels M, Carillon S, Desgrees du Lou A, Larmarange J (2020). Effect of organizational models of provider-initiated testing and counseling (PITC) in health facilities on adult HIV testing coverage in sub-Saharan Africa. AIDS Care.

[13] Marwa R, Anaeli A (2020). Perceived barriers toward provider-initiated HIV testing and counseling (PITC) in pediatric clinics: a qualitative study involving two regional hospitals in Dar-Es-Salaam, Tanzania. HIV AIDS (Auckl).

[14] Jin J, Yang H, Guo Z, L V X, Jiang X, Ding C (2023). Relationships of illness perception, symptoms response and social support with acute myocardial infarction patients’ prehospital delay in rural China: protocol for a cross-sectional study. BMJ Open.

[15] Wang B, Peng X, Liang B (2023). Loneliness and its correlates among older adults living with HIV: a multicenter cross-sectional study. J Affect Disord.

[16] Wang B, Peng X, Fu L (2023). Correlates of sexual lifestyles among older adults living with HIV in China: findings from the Sexual Well-being (SWELL) Study. Infect Microb Dis.

[17] Wang B, Peng X, Lu Y (2025). Association between frailty and low sexual function among sexually active older adults living with HIV in China: a multi-centre cross-sectional study in China. AIDS Care.

[18] Wang B, Peng X, Liang B (2023). Sexual activity, sexual satisfaction and their correlates among older adults in China: findings from the sexual well-being (SWELL) study. Lancet Reg Health West Pac.

[19] Wang B, Peng X, Liang B (2023). Sexual well-being among older adults in China (SWELL): protocol for a multicenter cross-sectional study. BMJ Open.

[20] Wang B, Peng X, Fu L (2024). Sexual function and correlates among adults aged 50+ years in China: findings from the sexual well-being (SWELL) study. J Am Geriatr Soc.

[21] Stover J, Glaubius R, Teng Y (2021). Modeling the epidemiological impact of the UNAIDS 2025 targets to end AIDS as a public health threat by 2030. PLoS Med.

[22] He N (2021). Research progress in the epidemiology of HIV/AIDS in China. China CDC Wkly.

[23] Tan K, Black BP (2018). A systematic review of health care provider-perceived barriers and facilitators to routine HIV testing in primary care settings in the southeastern United States. J Assoc Nurses AIDS Care.

[24] Naidoo N, Zuma N, Khosa NS (2018). Qualitative assessment of facilitators and barriers to HIV programme implementation by community health workers in Mopani district, South Africa. PLoS ONE.

[25] Lin B, Liu J, Ma Y, Zhong X (2022). Factors influencing HIV testing and counselling services among men who have sex with men in Western China: a cross-sectional study based on Andersen’s behavioral model. Environ Health Prev Med.

