# Supplementary material for: Perceptions, Barriers, and Facilitators of Provider-Initiated and Voluntary HIV Testing and Counseling Among Health Care Workers: Protocol for a Multicenter Cross-Sectional Study
Source: JMIR Res Protoc. 2025 Dec 22;14:e69832. doi: 10.2196/69832 (PMC12721219; doi:10.2196/69832)
Supplement: Multimedia Appendix 1 [file resprot-v14-e69832-s001.pdf]

Ethical Clearance Statement

This is to certify that the study titled *Study on barriers and facilitators in the implementation of HIV testing interventions from the perspective of providers*, which is being conducted through the survey titled *Provider-initiated views on provider-initiated HIV testing and counselling and voluntary HIV counselling and testing Study (PIVOT Study)*, will be carried out **Dr. Bingyi Wang** at the Guangdong Provincial Center for Disease Control and Prevention, and funded by the Guangdong Provincial Medical Science and Technology Research Foundation [B2025741] in 2025, involves the use of fully anonymized data.

In accordance with applicable ethical guidelines and regulations, studies that utilize only anonymized information are eligible for exemption from ethical review. This exemption is hereby confirmed and granted.

Medical Ethics Review Committee

Guangdong Provincial Center for Disease Control and Prevention

May 16, 2025
